# Identify future epidemic threshold and intensity for influenza-like illness in Iraq by using the moving epidemic method

**DOI:** 10.1016/j.ijregi.2023.12.005

**Published:** 2023-12-21

**Authors:** Hanan Abdulghafoor Khaleel, Riyadh Abdulameer Alhilfi, Salman Rawaf, Celine Tabche

**Affiliations:** 1 Communicable Diseases Control Centre, Directorate of Public Health, Ministry of Health, Baghdad, Iraq; 2 Directorate of Public Health, Ministry of Health, Baghdad, Iraq; 3 WHO Collaborating Centre, Department of Primary Care and Public Health, Imperial College London, UK

**Keywords:** Moving epidemic method (MEM), Epidemic, Flu, Influenza, Influenza-like illness (ILI), Preparedness

## Abstract

**Objectives:**

Influenza-like illness (ILI) entered the Iraq surveillance system in 2021. The alert threshold was determined using the cumulative sum 2 method, which did not provide other characteristics. This study uses the moving epidemic method (MEM) to describe duration and estimate alert thresholds for ILI in Iraq for 2023-2024.

**Methods:**

MEM default package was used to estimate influenza 2023-2024 epidemic thresholds. Analysis was repeated using optimum parameter of epidemic timing for fixed criteria method, which is 3.3. Arithmetic means and 95% confidence interval upper limit were used to estimate threshold. Geometric mean and 40%, 90%, and 97.3% confidence interval upper limits were used to estimate intensity levels. Aggregated Centers for Disease Control and Prevention surveillance data were used to detect epidemic thresholds, length, sensitivity, and predictive values.

**Results:**

ILI activity starts at week 30 and lasts 7 weeks. Optimized epidemic threshold is 4513 cases, lower than default (4540 cases). Optimized medium-intensity level was higher than default, and high and very high-intensity levels were lower.

**Conclusions:**

MEM is essential to determine an influenza epidemic's threshold and intensity levels. Despite requiring 3-5 years of data, using it on data for 2.5 years has resulted in an epidemic threshold slightly higher than the threshold calculated using the cumulative sum 2 method.

## Highlights

• Utilizing moving epidemic method (MEM), this study refines the understanding of influenza-like illness season.
• MEM identifies onset at week 30, lasting 7 weeks for public health planning.
• MEM outperforms traditional methods with a 6% increase in sensitivity.
• MEM has the potential to guide early public health interventions.
• MEM can support targeted measures like vaccination campaigns.

## Introduction

Influenza-like illness (ILI) is still one of the significant public health challenges worldwide, with yearly estimated new infections of one billion and further deaths of 290,000-650,000 [1,2]. ILI, also known as flu-like syndrome, is a group of infectious diseases caused by bacteria and viruses that result in a list of common symptoms. This is why it was moved from being a specific disease into a syndromic cluster for keeping track of the list of syndromes for analysis [3]. ILI's case definition is often described as fever (>38°C) with sore throat or cough; moreover, it might show up as a broader range of symptoms that include shivering, dry cough, body aches, nausea, loss of appetite, and sneezing [4]. As listed by Spencer et al. [4], in 2022, the most common pathogens for ILI are influenza viruses, common cold viruses [rhinovirus, adenovirus, human respiratory syncytial virus, parainfluenza virus, human metapneumovirus, and human coronavirus.

Despite the recent advances in diagnostics and modeling approaches to influenza, accurately estimating its public health impact and epidemic characteristics is still one of the complex challenges [5], [6], [7]. Modeling and surveillance strategies are used to boost prevention strategies.

Globally, influenza surveillance systems are the most intricate due to the spectrum of flu symptoms ranging from asymptomatic to severe, different reporting mechanisms in different countries, and various diagnostic approaches. For instance, some systems depend only on cases tested for influenza in the laboratory, while others include ILI and acute upper respiratory infections. In addition, ILI symptoms and clinical presentation overlap with pneumonia, upper respiratory tract infections, and COVID-19. Recent advancements in biostatistical methods to estimate epidemic thresholds and other characteristics of epidemic waves have facilitated early detection, thereby enacting public health measures in time to control the epidemic spread. An epidemic threshold, also known as basic reproduction number (R_0_), is a parameter in epidemiology that determines whether an infectious disease can spread throughout the population [8]. It is used to assess the potential risk of an epidemic to start implementing appropriate control measures. Nevertheless, variable surveillance systems and variable methods to estimate the thresholds made it challenging to compare countries. The most recent approach to estimating influenza thresholds and characterizing influenza epidemics is using the moving epidemic method (MEM). A method has recently been used in several countries, including Arab nations [9], [10], [11], as the standard for estimating influenza thresholds and intensity levels [12], [13], [14].

Optimal characterization of influenza seasonality requires data for at least 3-5 years to accurately estimate the epidemic thresholds and duration [13]. However, ILI entered the surveillance system in Iraq in 2021 when the case definitions for ILI, pneumonia, and COVID-19 were all updated, and all governmental surveillance sites started reporting weekly. Therefore, the surveillance system in Iraq applied the cumulative sum by groups to estimate the alert threshold of ILI/pneumonia to account for the few data available.

An extensive literature search showed that the MEM model had not been used previously in Iraq to estimate thresholds and determine intensity levels. Therefore, this study aims to describe the timing and duration of ILI and evaluate the alert thresholds in Iraq for 2023-2024 using the MEM model. In addition, this study aims to establish influenza intensity levels for 2023-2024 using the MEM model to guide influenza vaccination policy and other non-pharmaceutical public health interventions.

## Methods

### Data sources

Data regarding weekly reported aggregated cases of ILI were extracted from the National Surveillance System EpiInfo 7.2 template. The national surveillance system collects aggregated and case-based data from 20 Departments of Health (DsoH) representing all governorates with three DsoH in Baghdad: Karkh, Resafa, and medical city.

Aggregation is by age group, sex, district, and health facility for 13 syndromes or diseases, including ILI. The weekly report should be submitted within four working days after the end of the epidemiologic week. Age groups are divided into less than 5 years, 5-16 years, and more than 16 years.

### Case definitions

The national surveillance system added the following case definitions to the list of diseases since 2021:

- ILI: Any person with an acute respiratory infection characterized by a fever of ≥38°C and cough, with onset within the last 10 days. This means that ILI patients attended the outpatient clinic, did not have chest X-ray or computed tomography scan done, and had not been admitted to the hospitals.

### Study population

All residents of Iraq who attend governmental hospitals and main primary health care centers for ILI should be reported to the national surveillance system in the aggregated weekly surveillance forms. The primary healthcare centers are managed by physicians. The branched healthcare centers usually refer the cases or send their reports to the primary healthcare center, which combines the report and sends it to the district. Patients who attend the private sector are omitted.

### Statistical analysis

We used the retrospective national surveillance weekly data of ILI from 2021-2023 to detect the seasonality and set the epidemic and intensity thresholds for the upcoming season 2023-2024.

The Health Sentinel Network of Castilla y León (Spain) developed MEM as a standardized tool to help set the baseline of influenza activity and establish an epidemic threshold above which the activity parameter is considered in an epidemic period [5,14]. Moreover, MEM computes the influenza intensity thresholds using the highest values of each epidemic period combined and calculates the geometric mean and the upper limit of one-sided confidence intervals at 40% (medium), 90% (high), 97.5% (very high) of the n (n = 30/ number of seasons) highest epidemic weekly count [5,14]. Baseline is when the weekly count is lower than the epidemic threshold. The epidemic timing was calculated using the fixed criterium method, in which the default parameter was 2.8%. Optimization of the model resulted in a parameter of 3.3%, which was used in the final model. Estimates were rounded up as we used the number of cases rather than proportions.

The cumulative sum 2 method is the average of the 7 days prior till 2 days after the day we want to calculate the threshold for plus 1.96 standard deviation for the same period [15,16].

Levels of sensitivity, specificity, positive predictive value, and negative predictive value were also calculated through an iterative process and cross-validation procedure to assess the goodness of the default and optimized models. The process was repeated for each season in the dataset, and all true positives (TPs), true negatives (TNs), false positives (FPs), and false negatives (FNs) were pooled. TPs were defined as values of the epidemic period above the threshold, while TNs were values of the non-epidemic period below the threshold. FPs were values of the non-epidemic period above the threshold, and FNs were values of the epidemic period below the threshold.

Sensitivity reflects the number of epidemic weeks above the pre-epidemic threshold and above the post-epidemic threshold divided by the number of epidemic weeks (epidemic length). Specificity demonstrates the number of non-epidemic weeks below the pre-epidemic threshold and below the post-epidemic threshold divided by the number of non-epidemic weeks.

Positive predictive value reflects the number of epidemic weeks above the threshold divided by the number of weeks above the threshold. A negative predictive value reflects the number of non-epidemic weeks below the threshold divided by the number of weeks below the threshold.

Matthew's correlation coefficient and Youden's index were used to identify how well both the default and the modified models were in describing the ILI season in determining the epidemic threshold, post-epidemic threshold, and intensity levels [17].

### Software

We used the moving epidemic method R package, version 2.17 in RStudio 2022.12.0+353 “Elsbeth Geranium” Release (7d165dcfc1b6d300eb247738db2c7076234f6ef0, 2022-12-03) to get the estimates of epidemic thresholds and intensity thresholds at the national level.

### Ethical considerations

Approval of the research scientific and ethical committee was obtained from the Ministry of Health in Iraq. Case-based data were deidentified and used for statistical analysis only.

## Results

**Figure 1:**
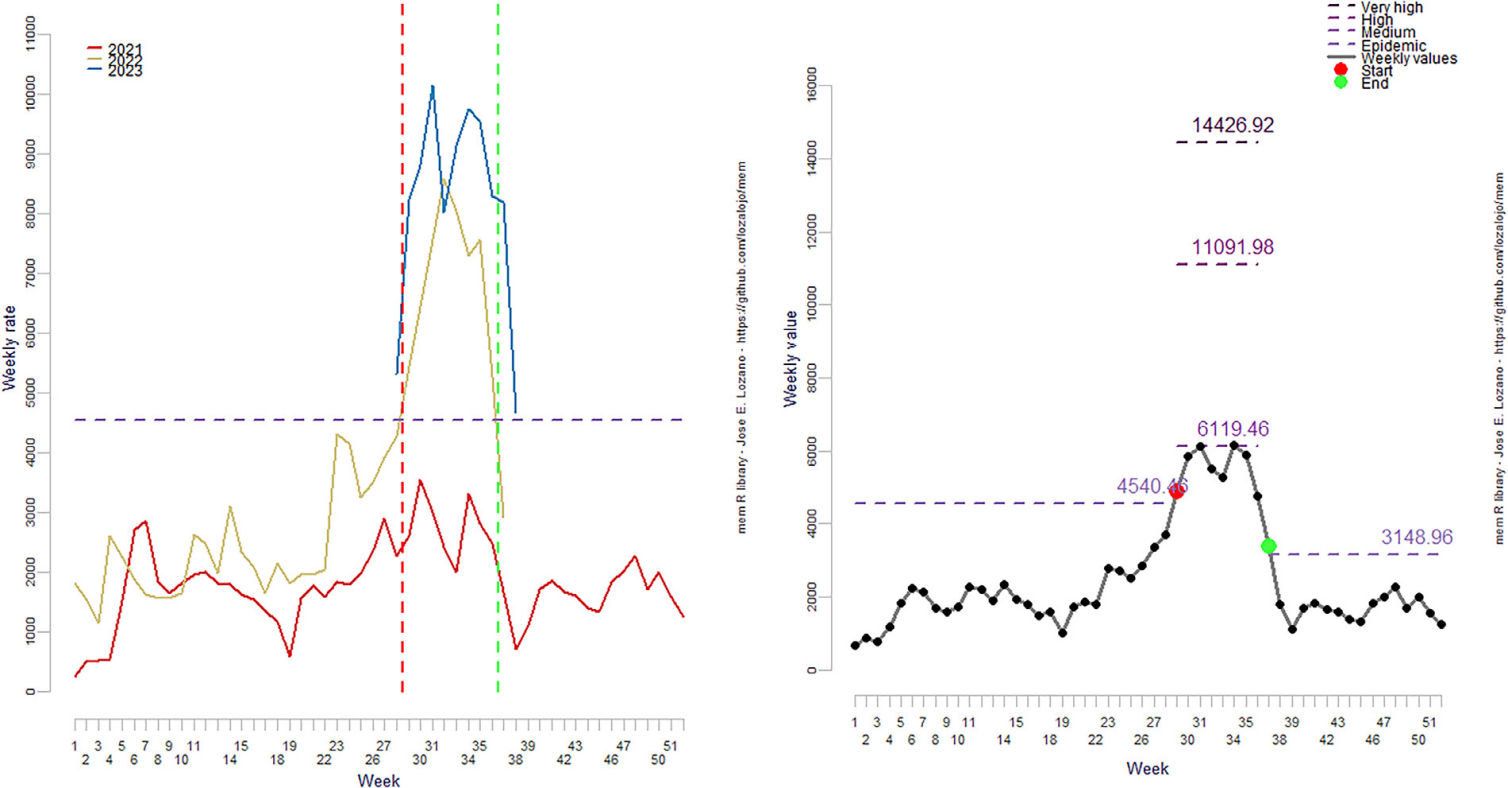
(a) Centered weekly values and the epidemic threshold. (b) Average weekly value and intensity levels. Epidemic threshold and intensity levels were calculated based on the default setting.

**Figure 2:**
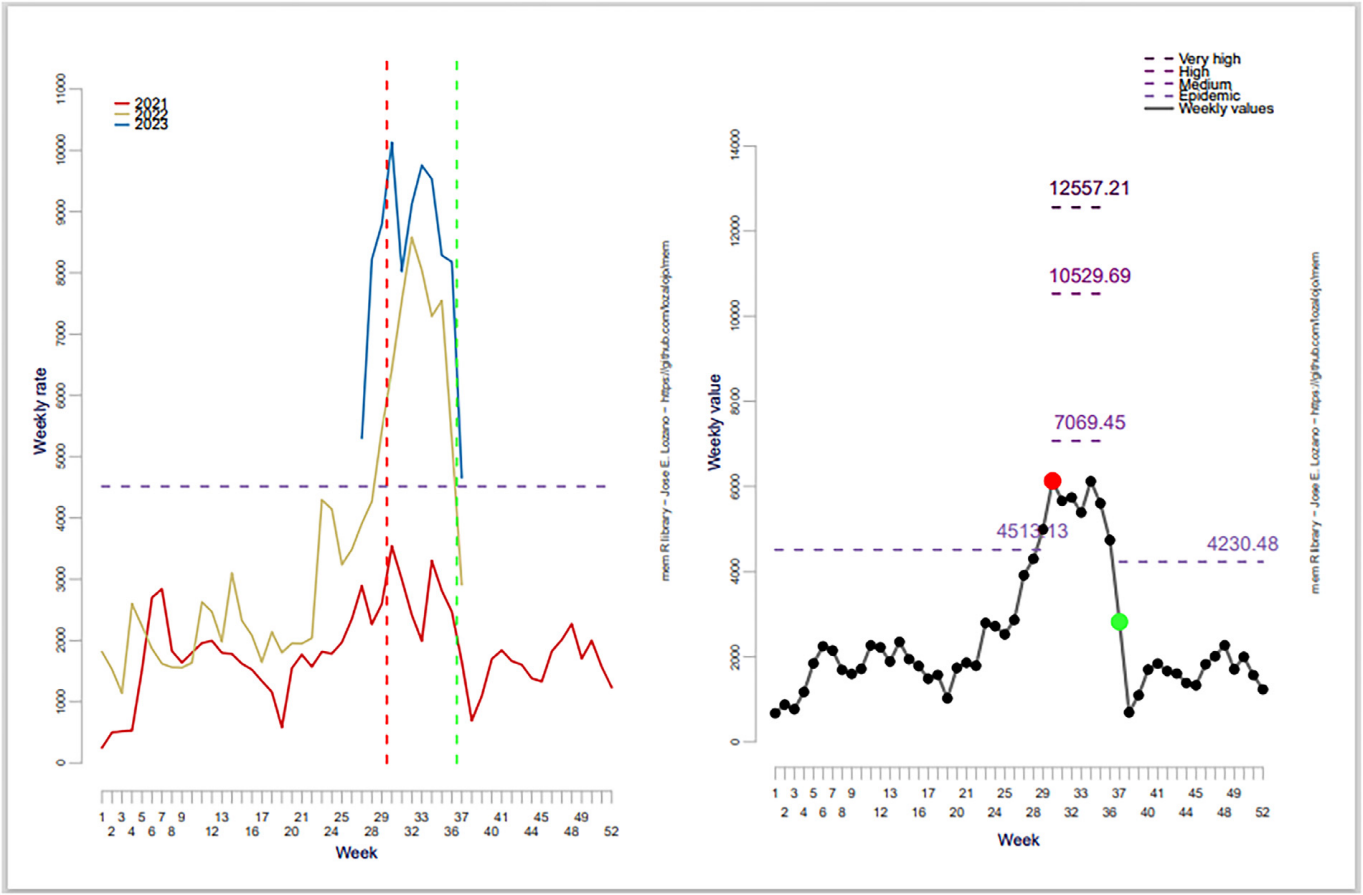
(a) Centered weekly values and the epidemic threshold. (b) Average weekly value and intensity levels. Epidemic threshold and intensity levels were calculated based on the optimum value.

Using the optimized approach, ILI activity usually starts at week 30 and lasts about 7 weeks. The optimized epidemic threshold is 4513 cases, slightly lower than the default (4540 cases) (Figures 1 and 2). The optimized medium-intensity level was higher than the default. In contrast, the high and very high-intensity levels were lower, as shown in Table 1.

**Table 1:** Epidemic start week, length, threshold, intensity levels, and goodness of the model for ILI, 2021-2023.

	No optimization	Optimization
**Epidemic start week, length, threshold, and intensity levels for ILI, 2021-2023**		
Seasons in the model	3	3
Average epidemic start week	29, 95% CI: [1,44]	30, 95% CI: [1,44]
Average epidemic length	8 weeks, 95% CI: [3,11]	7 weeks. 95% CI: [1,11]
Post-epidemic level	3149	4231
Epidemic percentage	33.6%	30.5%
Epidemic threshold	4540	4513
Medium threshold	6120	7070
High threshold	11,092	10,530
Very high threshold	14,427	12,557
**Goodness of the model**		
Sensitivity	0.66	0.72
Specificity	0.82	0.83
Positive predictive value	0.55	0.56
Negative predictive value	0.88	0.90
Percent agreement	0.78	0.80
Matthew's correlation coefficient	0.45	0.51
Youden's Index	0.48	0.55

ILI, influenza-like illness.

The average of the cumulative sum method 2 for ILI in 2022-2023 is 4023.

ILI cases during 2021 were lower than the alert threshold calculated through MEM, with a peak in week 29. When comparing the ILI cases in 2022 and early 2023, ILI cases started to rise in week 44 and declined for only week 52, which coincides with the Christmas celebrations rising again above the threshold.

**Figure 3:**
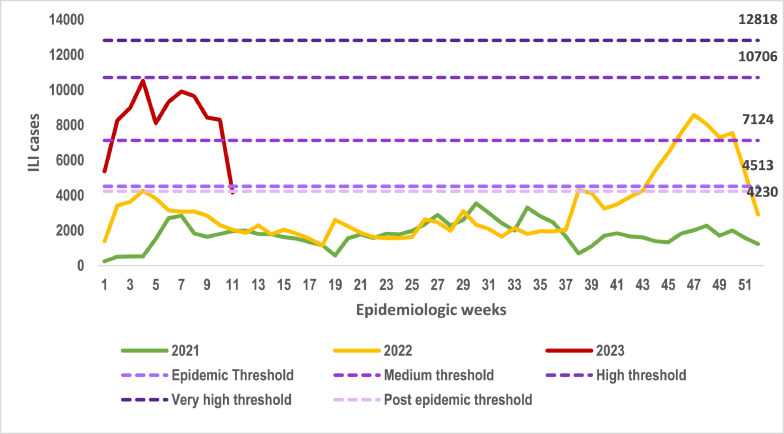
ILI values from 2021 to 2023 compared to default intensity levels, epidemic threshold, and post-epidemic threshold. ILI, influenza-like illness.

**Figure 4:**
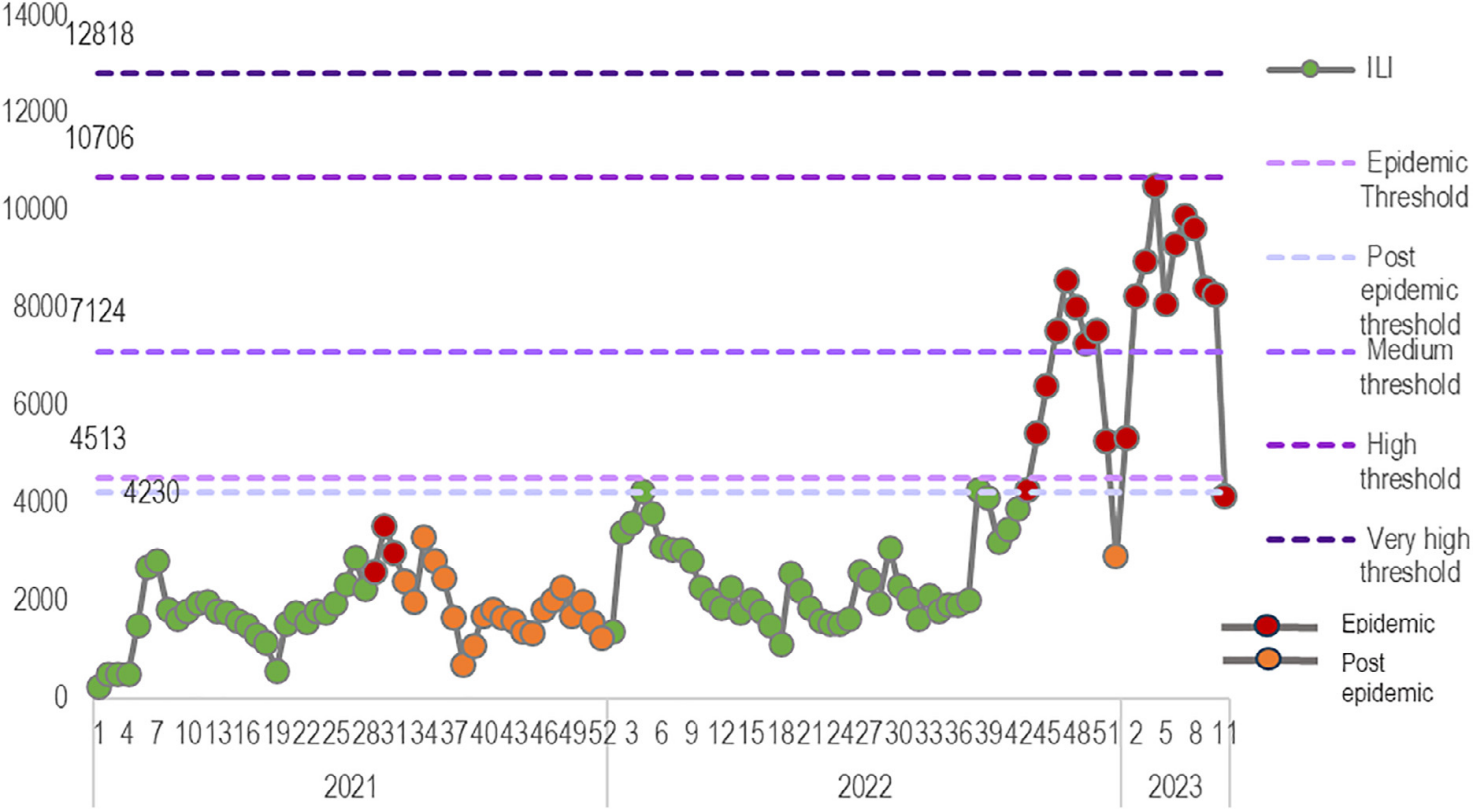
ILI seasons from 2021 to 2023 compared to default intensity levels, epidemic threshold, and post-epidemic threshold. ILI, influenza-like illness.

ILI season of 2023 had higher levels than the previous two seasons. Cases dropped suddenly in week 52, 2022, and then bounced back to the usual level reported for that season, as shown in Figures 3 and 4. All values reported in 2023 were at the medium-intensity level.

As for the goodness of the model, the sensitivity of the optimized model (0.72) was 6% higher than the sensitivity of the default model (0.66). The specificity, positive, and negative predictive values improved by only 1-2%, as shown in Table 1. Both Matthew's correlation coefficient and Youden's index improved by 6-7% in the optimized model.

## Discussion

This is the first application of the MEM model in Iraq to characterize ILI threshold and seasonal characteristics using national surveillance ILI data. Estimates from the model show an epidemic threshold of 4513, an average epidemic start week of 30, post-epidemic threshold of 4231, and an average duration of 7 weeks. The epidemic period forms 30% of the surveillance season.

Results from this study showed that the epidemic season usually starts in weeks 29-30 in late July and lasts for 8 weeks and ends in week 38 (late September) [18]. However, ILI cases were not tested in a laboratory and may have been due to COVID-19 as it was the circulating virus during that time. The epidemics lasted about 7-8 weeks, less than the average duration estimated from other studies [5,9,11,12,19,20], which could be due to limited data seasons and variable data points. The epidemic threshold obtained from the MEM model was slightly higher than the currently used cumulative sum 2 method, which facilitates using the value of the cumulative sum method as an alert to initiate public health measures to control ILI spread no matter what the causative agent is.

Levels of ILI were below the epidemic threshold in 2021 and 2022, which could be due to the public health measures undertaken to control the spread of COVID-19. As ILI and other acute respiratory infections all share the same mode of transmission, their circulation was also interrupted due to these measures.

In the northern hemisphere, the peak influenza activity is usually between week 40 and week 20 in the next year. In the Eastern Mediterranean Region, the influenza season usually lasts for 34 weeks, starting from week 36 (early September) to week 18 (early May). In contrast, the peak influenza activity in Iraq during the 2022-2023 season was between week 29 and week 37, which is earlier than has been found in other studies [11,12,21,22]. This might have been due to the relaxation of travel restrictions and increased travel and tourism activities in the summer of 2022. In addition, religious mass gathering called “Ashuraa” happened at the end of July as well that attracted a lot of visitors and occurs annually. Despite the moderate certainty of the model in characterizing the model, these findings suggest the necessity of early initiation of preparedness activities to secure vaccines and medications as well as raise awareness of the population and health care workers to ILI and the importance of implementing infection control practices to control its spread. This has been also highlighted by the epidemiological pattern of seasonal influenza in Iraq [23].

The model's sensitivity and specificity were moderate, which could be due to the few years of data available. Using the optimized model slightly improved the goodness measures. Nevertheless, the epidemic threshold resulting from the MEM model was very close to the one obtained from the cumulative sum 2 method currently used in surveillance. This indicates that the model did not produce significantly different measures and that the few data points may not have severely affected its performance. Having very close estimations from both models encourages using the MEM model as it can result in better estimates as more data becomes available.

Using this method in the future will help better describe ILI season and, possibly, other acute respiratory infections to help more directed planning of immunization policy, implementing non-pharmaceutical public health interventions, and distributing resources to reduce morbidity and mortality.

This study was mainly limited by a lack of ILI data before 2021, which could have underestimated the thresholds and intensity levels. In addition, intensity levels reflect the severity of the season rather than the severity of cases. Therefore, the surveillance may benefit from using the MEM model on severe acute respiratory infections to assess the season's severity and help in hospital preparedness activities.

## Conclusion

Applying the MEM method to estimate the threshold and intensity levels at the subnational and district levels to monitor ILI activity during 2023-2024 will strengthen surveillance. Furthermore, using this method for other communicable diseases such as pneumonia, COVID-19, influenza, and its types, as well as acute diarrhea, and comparing the values with the cumulative sum to detect which is most sensitive in early detection of outbreaks. This study indicates that using the MEM as the standard method for determining ILI thresholds and intensity levels as more data become available should lead to more studies and research testing its effectiveness in the future. The epidemic threshold calculated using the cumulative sum 2 method could be an alert threshold instead for preventive control measures to commence.

## Declarations of competing interest

The authors have no competing interests to declare.
